# The Effects of Aquatic Exercises on Physical Fitness and Muscle Function in Dialysis Patients

**DOI:** 10.1155/2015/912980

**Published:** 2015-06-16

**Authors:** Wioletta Dziubek, Katarzyna Bulińska, Łukasz Rogowski, Tomasz Gołębiowski, Mariusz Kusztal, Monika Grochola, Dominika Markowska, Agnieszka Zembroń-Łacny, Wacław Weyde, Marian Klinger, Marek Woźniewski

**Affiliations:** ^1^Department of Physiotherapy, University School of Physical Education, 35 Paderewskiego Street, 51-612 Wrocław, Poland; ^2^Non-Public Medical College of Wroclaw, 69 Nowowiejska Street, 50-340 Wrocław, Poland; ^3^Department and Clinic of Nephrology and Transplantation Medicine, Wroclaw Medical University, 213 Borowska Street, 50-556 Wrocław, Poland; ^4^Department of Biological Basis of Sport, University of Zielona Gora, 58 Wyspianskiego Street, 65-178 Zielona Gora, Poland

## Abstract

*Purpose*. The aim of this study was to assess the impact of a 3-month physical training program, conducted in an aquatic environment with end-stage renal disease patients (ESRD), on the physical fitness and functional parameters of the knee joint muscles. *Patients and Methods*. The study included 20 ESDR patients with mean age 64.2 ± 13.1 y. treated with hemodialysis in Dialysis Center of the University Hospital in Wroclaw. Before and 3 months after the physical training in water, a test was performed to evaluate the physical fitness of each patient; additionally, a measurement was taken of force-velocity parameters. The 3-month training program took place on nonhemodialysis days, in the recreational pool of the University of Physical Education in Wroclaw. *Results*. After aquatic training cycle, an improvement was observed in all parameters measured using the Fullerton test. The value of peak torque and its relation to body mass increased in the movement of flexors and extensors of left and right lower extremities in all tested velocities. *Conclusions*. In assessing the physical fitness of studied women, the biggest improvement was achieved in tests assessing the strength of upper and lower extremities as well as lower body flexibility. Higher values of force-velocity parameters are conducive to women achieving better physical fitness test results.

## 1. Introduction

Chronic kidney disease (CKD) is a syndrome that evolves as a result of progressive and irreversible impairment of renal function. End-stage renal disease (ESRD) or CKD stage V is characterized by structural and functional damage to the kidneys (loss of glomerular filtration) resulting in many metabolic disturbances, due to accumulation of waste products in the blood which are toxic to the body. In ESRD any form of renal replacement therapy (kidney transplantation, hemodialysis, or peritoneal dialysis) must be started. Hemodialysis treatments are most frequently chosen in developed countries. Standard chronic hemodialysis program consists of 3 times per week sessions with a duration of 4 to 6 hours, the length of which is determined individually depending on the patient's condition.

The ongoing nature of the disease and the lengthy of lifelong renal replacement therapy are factors that significantly deteriorate the physical fitness of patients with CKD. Patients with ESRD undergoing hemodialysis treatments have a significantly reduced exercise tolerance, exercise capacity, strength, and endurance compared to healthy individuals and patients with a lighter form of the disease, who do not require dialysis treatments [1, 2].

Most dialysis patients lead a sedentary lifestyle and are functionally limited due to deteriorating health. It should be noted that the hemodialysis treatment itself takes place in a supine or semisitting position from 4 to 6 hours per visit, which adds up to around 400 to 900 hours per year without any physical activity. A low level of physical fitness is associated with significant impairment of daily activities, including those related to self-care (e.g., bathing, housework, dressing, and shopping), paid work, functioning in the community, and recreation [3, 4]. It is unknown to what extent limitations in physical functioning are inevitably a result of renal failure and/or dialysis treatment and to what extent a result of reduced physical activity. We do know, however, that a reduction in daily physical activity lowers the quality of life of the patient and that it is an independent predictor of mortality [3–6].

Patients on hemodialysis who lead a sedentary lifestyle are exposed at a risk of mortality by 62% per annum compared to physically active patients [7]. It is estimated that each month of dialysis reduces their physical activity level by 3.4% [8].

Many studies have shown that patients on dialysis have weaker muscle strength and endurance than healthy individuals. This applies to both the phasic and postural muscles [1, 8–11]. The causes of muscle weakness are complex and have not been fully elucidated. The main reasons for the reduced muscle strength and endurance are loss of muscle mass, atrophy of both types of fibers (especially type II), decline of the ability to generate force per unit of mass (myopathy), and decrease in the motoneurons activity [1, 9–12]. This also leads to a reduction in the muscle capillarization [11]. Structural changes within the ailing muscles, resulting from CKD, translate into functional changes, including changes in muscle strength, muscle endurance, and activity of muscle ergoreceptors, which is an indication that regular exercise needs to be undertaken, even by patients with end-stage renal failure.

The effects of regular physical exercise of moderate-intensity performed during or between dialysis treatments have many physiological and functional benefits [3, 4, 13–15]. Regardless of whether the physical training is performed on a nondialysis day or during the first two hours of dialysis treatment, it leads to an improvement in aerobic capacity, resulting in, among other positive effects, an increase in left ventricular ejection fraction (LVEF), a decrease in blood pressure, and modification of other risk factors [16]. An adaptation to physical exercise also causes skeletal muscle hypertrophy (increases in surface area of fibers type I as well as fibers types IIa and IIx in cross-section) [12] and subsequently leads to improved muscular strength, power, reduction in the level of fatigability, and an overall improvement in physical fitness of patients with end-stage renal disease [17]. Their quality of life and daily functioning also improve [18].

Introducing endurance and strength training to a rehabilitation program for patients on hemodialysis provides various health benefits. Aerobic training increases insulin sensitivity, improves lipid profile, raises hemoglobin concentrations, leads to increased endurance, lowers blood pressure, and improves quality of life. Resistance training, however, improves muscle strength, increases the level of physical fitness, and causes elevated concentrations of insulin-like growth factor 1 (IGF-1) to decrease, particularly when accompanied by persisting acidosis and the use of a low-protein diet [19, 20].

A combination of endurance-strength training is possible under aquatic conditions, in which water features like buoyancy and resistance are used with a minimal risk of musculoskeletal injury. Exercises in water are therefore a safe form of physical activity for people with multiple illnesses, the effectiveness of which is confirmed by research results [21].

In the literature, there are only a few studies on health benefits gained from aquatic exercises in patients with chronic renal failure [22–24]. Therefore, the purpose of this work is to assess the impact of a 3-month physical training program, conducted in an aquatic environment with end-stage renal disease patients, on the physical fitness and functional parameters of the knee joint muscles.

## 2. Material and Methods

### 2.1. Patients Characteristics

The study included 20 ESDR patients (16 females and 4 males) with mean age 64.2 ± 13.1 y. treated with hemodialysis in Dialysis Center of the University Hospital in Wroclaw.

Review of medical contraindication in all patients in hemodialysis was maintained in Dialysis Center of University Hospital; *n* = 86. 30 patients were excluded due to dementia, disability or leg amputation, deafness, blindness, heart failure, skin dermatitis, skin wound/hematoma, pleural effusion, recurrent infections, or severe malnutrition.

Inclusion study criteria were arteriovenous fistula as vascular access for hemodialysis (permanent central catheter was considered as contraindication), patient being able to reach swimming pool and to swim, stable clinical condition (controlled hypertension, no congestion or edemas, and no chest pain), and acceptable parameters of dialysis adequacy. 40  patients met inclusion criteria and were proposed to participate in the study. Finally, 20 of them gave informed consent and were enrolled in the study.

In order to carry out the study, an approval from the Bioethics Committee of the University of Physical Education in Wroclaw was obtained. All patients gave their informed written consent to participate in the study.

Hemodialysis treatment period before the program ranged from 4 to 174 months, 42.3 ± 6 months on average.

A list of causes of chronic renal failure in the study group is presented in Table 1.

Patients were informed at the beginning that they may opt out of exercises at any stage without giving a reason, and that is why the dropout rate is 35%. There was one death in study group (5%) unrelated to physical training. A full cycle of 3 months of physical training in water was completed by 12 women out of 20 persons entering the program (60%).

Before and 3 months after the physical training in water, a test was performed to evaluate the physical fitness of each patient; additionally, a measurement was taken of force-velocity parameters. We performed these tests at the Laboratory of Functional Studies in Internal Medicine of the University of Physical Education in Wroclaw. The 3-month physical training program took place on nonhemodialysis days, in the recreational pool of the University of Physical Education in Wroclaw (measuring 16.5 m by 4.5 m with a depth of 0.9 m). Training was performed in groups.

### 2.2. Description of Aquatic Exercises

Physical aquatic training was conducted in water for a period of 3 months, once a week for 60 minutes at a time. It was in the form of specialized gymnastics in water with music, using various types of gear (including foam tubes, buoyancy belts, foam dumbbells, and gloves). The training consisted of a warm-up, the main part (including endurance exercises, exercises strengthening particular muscle groups, and coordination exercises), and the end part, which consisted of stretching, breathing, and relaxation exercises. Withdrawal from exercise took place in the case of a patient feeling unwell or tired, experiencing nausea, vomiting, shortness of breath, dizziness, muscular, joint, or coronary pain. During the training, the participants were under constant supervision of a physiotherapist, a doctor, and a lifeguard.

## 3. Study Methods

The respondents' physical fitness was assessed on two occasions by the Fullerton Functional Fitness Test by Rikli and Jones, whereas the force-velocity parameters were taken using functional dynamometry in isokinetic conditions.

### 3.1. Fullerton Functional Fitness Test by Rikli and Jones (Senior Fitness Test)

The Fullerton test assesses functional capacity of the elderly and patients undergoing a process of rehabilitation. It provides an opportunity to assess the level of basic motor skills: strength, flexibility, coordination, and physical endurance, which are evaluated in 6 motor tasks, carried out in the following order.

1. Arm curl is an indirect test evaluating the strength of the upper body. The result of the test comprises the number of bends made with supination of the dominant forearm, holding a hand weight of 8 lbs (for men) and 5 lbs (for women), during a period of 30 seconds in a seated position on a chair without backrest.
2. Chair stand is an indirect test evaluating the strength of the lower body. The result of the test comprises the number of rises made from the chair, with arms across the chest to a full upright position, during a period of 30 seconds.
3. Back scratch is an indirect test evaluating the flexibility of the upper body. A measurement is made using a 30 cm ruler to determine the distance between the middle finger of the dominant hand placed on the top of the back (fingers pointing down) and the middle finger of the nondominant hand placed on the bottom of the back (fingers pointing upward).
4. If the fingertips touch then the score is zero. If they do not touch, measure the distance between the finger tips (a positive score); if they overlap, measure by how much (a negative score). Practice two times, and then test two times, selecting the best result. Stop the test if the subject experiences pain.
5. Chair sit-and-reach is an indirect test evaluating the lower body flexibility. A measurement is made using a ruler, to determine the distance between the tip of the fingertips and the toes. If the fingertips touch the toes then the score is zero. If they do not touch, measure the distance between the fingers and the toes (a negative score); if they overlap, measure by how much (a positive score). The test is performed twice, selecting the best result.
6. Eight-foot up and go is an indirect test evaluating the motor agility and dynamic balance in conjunction with the respondent's balance. A measurement is made of the shortest possible time it takes the respondent to rise from a chair, walk around a cone placed at a distance of 8 foot, return to his or her chair, and take a sitting position. The test is performed twice, selecting the best result.
7. A 6-minute walk test (6MWT) is an indirect test evaluating the level of exercise capacity. The outcome of the test comprises the distance covered along a marked 30-meter corridor in 6 minutes at a marching pace: one that the respondent uses daily. Prior to the test, the respondent is informed of the possibility of stopping for a moment if needed during the test. The test is discontinued when the respondent reports dizziness, occurrence of nausea, extreme fatigue, pain, or alarming symptoms noticed by the researcher. For the subjective assessment of fatigue, a 10-point Borg scale was used (where 0 means no fatigue or dyspnea, and 10 indicates maximum fatigue or dyspnea) (ATS Statement, 2002). Prior to commencement of the Fullerton test and after trials 1, 2, and 6, measurements of hemodynamic parameters of blood pressure and heart rate were made using an arm-type electronic sphygmomanometer. Before commencing these tests, subjects were given specific instructions; in addition, each test was preceded by a demonstration [25–28].

### 3.2. Assessment of Muscle Strength of the Lower Extremities in Isokinetic Conditions

**Figure 1:**
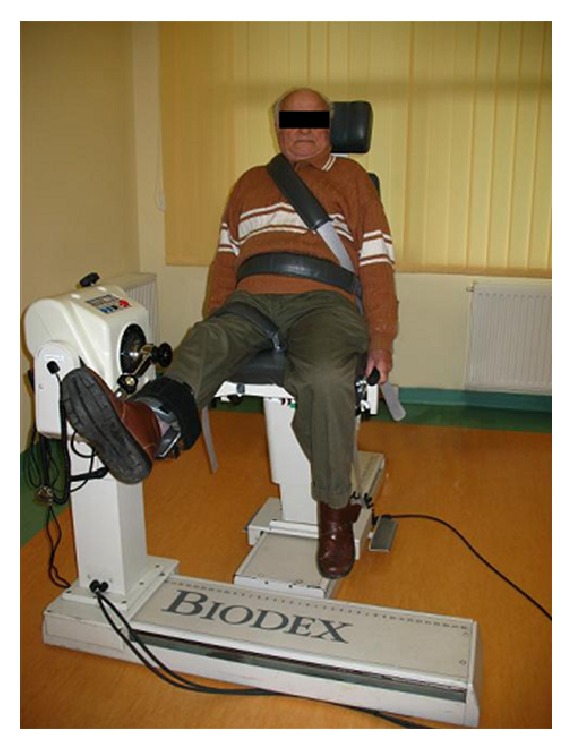
Patient using Biodex station during the test (own source).

Studies of the force-velocity parameters were performed using the Biodex Multi-Joint System 3 isokinetic dynamometer (Figure 1). An assessment was made of the functionality of flexors and extensors of the knee joint.

Before each test, the seat, dynamometer, and a suitable knee attachment were adjusted so that the tip of the dynamometer became an extension of the axis of rotation in the examined joint. For all respondents, the same range of flexion and extension of the knee joint was established at 90° (S 0-0-90), with an allowance for gravity adjustment. The thigh and pelvis of a patient were stabilized using straps attached to the chair so as to eliminate movements in neighboring joints. A starting position for the test was a maximal flexion of the lower extremity at the knee joint.

The test consisted of a warm-up—the subject performed 3 submaximal flexion and extension movements in each knee and 1 maximum movement in order to become familiar with a given load—and the main part, which involved measuring peak torque (Nm) at preset angular velocities, respectively, 60°/s, 180°/s, and 300°/s.

With the angular velocity of 60°/s, respondents performed 5 repetitions, while at 180°/s and 300°/s they performed 10 reps. Muscle function parameters were recorded: peak torque [Nm], peak torque/body weight [%], total work [J], and average power [W]. There was a 60-second break between subsequent attempts. It was imperative for participants to exert maximum muscle strength in the shortest possible time for each movement [29–31].

## 4. Methods Used for Statistical Analysis

A basic statistical description of the analyzed material determined the mean values and standard deviation. The significance of changes in measured values (PT: peak torque, TW: total work, and AvP: average power) was assessed using Student's *t*-test for dependent samples. A relationship of change in the muscle strength and angular velocity of movement of the knee joint was determined using a nonparametric Friedman test [32].

An interdependence of the characteristics of physical fitness (results of Fullerton test) and the force-velocity parameters (BIODEX) was analyzed by determining Spearman's rank-order correlation coefficient-*ρ*.

## 5. Research Results and Discussion

The most common comorbidities that were identified in the study group were hypertension, which had prevailed in 18 patients (90%), ischemic heart disease (IHD) in 7 patients (35%), and peripheral artery disease of the lower extremities in 5 patients (25%) (Table 2).

### 5.1. Physical Fitness

The results of six Fullerton tests are shown in Table 3.

After a 3-month specialized aquatic training cycle, an improvement was observed in all parameters measured using the Fullerton test. The biggest increase was recorded in the “arm curl” and “chair stand” test trials, corresponding to the strength of upper and lower extremities. Flexibility of the lower part of the body also increased significantly (chair seat and reach, 1.5 cm further). An improvement was also achieved in the agility and dynamic balance of exercising respondents, at a borderline significance (*P* = 0.05). Subjects obtained a faster time by 1.22 seconds (average).

### 5.2. Force-Velocity Parameters of the Flexor and Extensor Muscles of the Knee Joint

After a 3-month cycle of aquatic training, the value of peak torque and its relation to body mass increased in the movement of flexors and extensors of left and right lower extremities in all tested velocities.

Most of the observed changes in torque are statistically significant; however, in the case of left extremity, these changes concern a movement performed at a velocity of 300°/s for extensors and a velocity of 180° and 300°/s for the flexor muscles (Table 4).

In isokinetic conditions, the lesser the angular velocity, the more the movement becomes resistive for the respondent, provoking muscles to generate maximum force. At a high velocity, the speed of executing movement increases with force, which indirectly determines the muscular strength/resistance being examined.

Analyzing the data, it can be concluded that as a result of a 3-month specialized aquatic training cycle, there has been a significant increase in the total work and average power of flexors and extensors of the knee joint in all measured velocities. Only in the case of the lower left extremity was there no significant change in the values of TW and AvP for the extensors at a velocity of 60°/s (*P* = 0.1535, *P* = 0.1794); similarly, for flexor muscles, there was no statistical improvement of AvP (*P* = 0.1074) (Table 4).

### 5.3. Peak Torque Gain of Flexors and Extensors of the Knee Joint at Different Angular Velocities

The process of changes in the average values of particular parameters, describing the torque of flexors and extensors of the knee joint, is presented in Table 5.

We observe that an increase in angular velocity causes a decrease in peak torque [N-m]. In the case of the lower right extremity, these changes are statistically significant, while in the case of the lower left extremity, these changes are slightly smaller. A significant relationship between torque with reference to body mass and angular velocity can only be determined in the case of knee joint extensors (*P* = 0.011). Changes in peak torque produced by the lower left extremity were insignificant. It should be noted, however, that the effect of diminishing muscle torque with increasing angular velocity has been observed in all tested values, and the lack of statistical significance in the left extremity is a consequence of a small amount of data.

### 5.4. Physical Fitness and Peak Torque

Table 6 shows Spearman's rank-order correlation coefficient-*ρ* between the results of individual Rikli and Jones test trials and peak torque at angular velocities of 60°/s, 180°/s, and 300°/s.

Higher correlations of statistical significance (and therefore stronger) in both examined velocities were found in tests that measured the following: balance and coordination (8-foot up and go test), strength of the lower extremities (chair stand), and exercise capacity/endurance (6MWT). At a velocity of 60°/s, PT correlates positively with the strength of upper extremities. Higher values of force-velocity parameters therefore contribute towards better test results of physical fitness in women.

## 6. Discussion

Chronic renal failure and prolonged or even lifelong processes of dialysis treatments cause deterioration of physical fitness in patients, which translates into their daily functioning and quality of life [3, 4, 18, 33]. Comorbid disorders are a common reason for deliberate reduction of physical activity by patients with ESRD, for fear of health deterioration. However, substantial research shows beneficial effects of a properly selected exercise program for this group of patients as an integral part of the rehabilitation process [5, 13]. The type of physical rehabilitation for patients with ESRD is associated with obtaining various physiological and functional benefits. Training where one unit encompasses both endurance and strength exercises gives more benefits than a one-track unit [19]. Physical training in an aquatic environment provides the opportunity to develop all motor skills; therefore, undertaking the problem of the impact of water exercises on the physical fitness of hemodialysis patients became a goal of this work.

Aquatic exercise was chosen since only data on beneficial effect on CKD (stage 2–4) patients (no dialysis population) were published (usually small groups) but no data on dialysis (high risk of cardiovascular event) patients were available. This mode of physical activity was chosen as the efficacy of physical exercises in aquatic environments has confirmed their cardioprotective effect in patients with CKD, including a reduction in systolic and diastolic blood pressure (hypertension in present in 90% of renal patient) as well as increased oxygen uptake.

Results of our own research confirm a significant impact of specialized aquatic training on the increase of physical fitness, especially in the strength of the extremities. This is a desirable effect of rehabilitation due to muscular atrophy, structural changes of muscle fibers, and accompanying neurodegenerative changes in the motor unit as well as atrophy of capillaries [1, 9, 11, 12].

Studies by Konstantinidou et al. [34] which evaluated the effectiveness of three rehabilitation programs of patients with ESRD—supervised exercises on days without dialysis, exercises during dialysis, and unsupervised exercises at home—showed dominance of the first program in achieving significant improvements in the body's aerobic fitness. In the literature on the subject, there have been assessments of physical exercise programs most often conducted on dialysis days and less frequently on days without dialysis, the frequency of which varies from two to three times a week with training cycle duration from three to six months [13, 16]. The functional benefits attained by the female patients who participated in a 3-month supervised aquatic training program once a week are confirmed by the results of this study, which indicate a high efficacy of the proposed training.

To date, studies evaluating the efficacy of physical exercises in aquatic environments have confirmed their cardioprotective effect in patients with ESRD, including a reduction in systolic and diastolic blood pressure, increased oxygen uptake (VO2max), and lower levels of both the urinary protein excretion rate (proteinuria) and levels of cystatin C, which indicate an improvement of renal function [22, 23]. As a result of the long-term regular aquatic training undertaken by patients with CKD, discontinuance of progression of the disease has taken place as well as a reduction in mortality rate in a 10-year observation period [24].

Ali et al. [35] measured the effect of swimming exercise (three days a week for 45 min) on adenine-induced CKD in nephrectomized rats. They observed that swimming exercise did not affect the salutary action of dietary supplement gum acacia on renal histology, but it partially improved some biochemical and physiological analyses, suggesting that addition of this mode of exercise may improve further the benefits of dietary supplementation of gum acacia.

The level of muscle strength and endurance measured by the functional dynamometry is a significant factor conditioning the physical capacity of the patient [36, 37].

Peak torque (PT) is considered the most important indicator of muscle strength. It can be used to identify early impairment of muscle performance as well as to evaluate the maximum level of muscular strength [36].

The total work (TW) is work performed by muscle groups throughout the entire test, indicating endurance capability of particular muscle groups. It is considered the most sensitive parameter for the assessment of muscle fatigue [36].

Maximum force, developed in a few seconds during the most intense workouts, is more useful than muscle strength as an indicator of the ability to perform dynamic efforts. It conditions the physical capacity of moving about and performing many daily tasks, especially in the elderly [37].

A three-month physical training program performed in an aquatic environment led to an improvement in almost all force-velocity parameters assessed at three angular velocities (60°, 180°, and 300°/s), in both the flexor and extensor muscles of the knee joint. Only in the case of the left lower extremity is the significance of test results not fully confirmed; this may be associated with habitual use of the dominant lower extremity, which in this case was the right extremity and because of a small number of respondents the functional improvement of this extremity was not significant.

In a study by Kouidi et al. [12], a 6-month rehabilitation of hemodialysis patients led to an increase in the proportion of type II fibers (by 51%) as well as an increase of the area of muscle fibers (by 29%) in the quadriceps (thigh) muscle. Changes have also been confirmed in the capillarization; moreover, the number of mitochondria has increased [12]. The results of these studies are supported by the increase in strength and muscular endurance, expressed as peak torque, average power, and total work, achieved after a 3-month rehabilitation program in an aquatic environment.

Confirmation of our research also follows research by Headley et al. [38], in which force-velocity parameters were measured using the Cybex Norm isokinetic dynamometer. After a 12-week cycle of resistance training in patients with ESRD, a significant increase was reported in peak torque at an angular velocity of 90°/sec (139.1 +/− 19.3 N-m), in addition, an increased distance was reported in the 6-minute corridor test (548.3 +/− 52.1 m), as well as a time reduction in performance of ten repetitions of “sit-to-stand-to-sit” test (17.8 +/− 1.9 sec) [38].

An improvement in physical fitness of hemodialysis patients was also shown in selected trials of the Fullerton test in a study by Painter et al. [18]. It led to a significant increase in the distance covered and acceleration of walking speed (6MWT test) as well as to an increase in strength of lower extremities measured in the “sit-to-stand” test [18]. In the results of our own research, a statistical significance of changes has been observed in all Fullerton tests; however, only the “arm curl,” “chair stand,” and “chair sit-and-reach” tests showed a high level of significance (*P* < 0.001).

The relationship between muscular strength and particular test trials that evaluate physical fitness can be helpful in determining the patient's level of functioning in everyday life. In our study, this relationship applies to strength of lower extremities, marching capacity, dynamic balance, and coordination of the studied women. Csuka and McCarty [39] have demonstrated a significant correlation of peak torque and muscle strength of lower extremities assessed by the “chair stand” test. This indicates that changes in muscle function translate into overall physical fitness in patients with chronic renal failure.

Findings on the effects of exercise in an aquatic environment on the health and functioning of dialysis patients show many positive changes; however, a small number of studies leave a significant gap in defining its scope. Our findings refer to the improvement of physical fitness in most investigated parameters; however, continuation of further studies is warranted, in order to further assess various aspects of life of patients with ESRD, including social functioning.

Twelve female patients who regularly exercised in water willingly took part in the classes.

The variety of exercises, use of attractive aiding tools, and physical activity in an aquatic environment have all contributed to the full involvement of respondents in the rehabilitation process. An additional motivating factor was the group nature of the activities, which contributed to the making of social contacts among the respondents.

Among patients with end-stage renal failure, the safety that the water offers during classes in a recreational pool is also an important factor in undertaking regular physical effort as was observed in this study. Both the endurance and strength aspects of water activities should be important elements of a comprehensive rehabilitation program for this group of patients.

The study patients were highly motivated to continue aquatic exercise (role of social and emotional factors).

## 7. Conclusions

1. After a 3-month physical training course in water, an improvement has been recorded in the force-velocity parameters and physical fitness of women on hemodialysis.
2. In assessing the physical fitness of studied women, the biggest improvement was achieved in tests assessing the strength of upper and lower extremities as well as lower body flexibility.
3. Higher values of force-velocity parameters are conducive to women achieving better physical fitness test results.

## Figures and Tables

**Table 1:** Summary of the causes of chronic renal failure in the study group.

Causes of chronic renal failure	Number of patients	%
Hypertensive nephropathy	10	50
Chronic glomerulonephritis	4	20
Interstitial nephropathy	2	10
Polycystic kidney disease	1	5
Diabetic nephropathy	2	10
Renal cortical necrosis	1	5

**Table 2:** Summary of comorbidities in the studied group.

Comorbidities	Number	%
Hypertension	18	90
Ischemic heart disease	8	40
Occlusive artery disease	5	25
Pacemaker	1	5
Artificial aortic valve	1	5
Mitral regurgitation	1	5
Atrial fibrillation	2	10
Stroke	1	5
Diabetes type 1	1	5
Diabetes type 2	2	10

**Table 3:** Results of Fullerton test before and after aquatic gymnastics.

Fullerton test	Before water exercises		After 3-month water exercises		Student's *t*-test	
	Mean	SD	Mean	SD	*t*	*P* value
Eight foot up and go [s]	7.02	3.02	5.80	1.48	2.240	**0.050**
Arm curl [n]	15.8	4.7	18.4	5.3	7.005	**<0.001**
Chair stand [n]	12.3t	4.1	15.5	4.8	6.550	**<0.001**
Back scratch [cm]	10.9	11.8	8.1	9.0	2.950	**0.016**
Chair seat and reach [cm]	5.5	2.4	4.0	2.6	5.582	**<0.001**
6-minute walk test [m]	345.0	101.0	424.5	76.0	3.185	**0.011**

**Table 4:** Values of force-velocity parameters of flexors and extensors of the knee joint before and after aquatic exercise.

Knee extensors								
Joint angle	Parameter	Leg	Before exercise		After exercise		Student's *t*-test	
			Mean	SD	Mean	SD	*t*	*P*
60°/s	Peak torque [N-m]	Right	62.31	16.55	68.84	15.25	4.621	**0.0024**
		Left	65.26	24.52	66.64	21.10	0.360	0.7293
	Peak torque/body weight [%]	Right	106.16	26.10	124.48	20.92	4.478	**0.0029**
		Left	109.33	36.13	120.16	25.21	1.347	0.2199
	Total work [J]	Right	375.73	98.88	438.56	83.89	5.695	**0.0007**
		Left	368.51	137.09	399.80	113.82	1.601	0.1535
	Average power [W]	Right	41.89	12.40	48.38	10.89	7.326	**0.0002**
		Left	41.96	15.67	45.85	13.46	1.492	0.1794

180°/s	Peak torque [N-m]	Right	44.44	10.66	49.13	9.13	4.727	**0.0021**
		Left	41.93	11.78	44.63	10.61	1.783	0.1178
	Peak torque/body weight [%]	Right	68.39	11.11	77.49	8.41	6.974	**0.0002**
		Left	65.14	15.37	69.81	13.04	1.984	0.0877
	Total work [J]	Right	531.39	145.57	607.19	123.65	4.760	**0.0021**
		Left	484.05	157.76	548.48	162.82	4.324	**0.0035**
	Average power [W]	Right	71.34	16.78	83.23	14.61	7.031	**0.0002**
		Left	67.38	21.63	74.90	22.29	2.974	**0.0207**

300°/s	Peak torque [N-m]	Right	38.39	3.57	39.94	3.62	4.116	**0.0045**
		Left	36.63	5.03	39.24	6.31	2.457	**0.0436**
	Peak torque/body weight [%]	Right	61.85	5.07	63.15	5.32	3.111	**0.0171**
		Left	60.16	11.99	61.24	12.10	0.422	0.6857
	Total work [J]	Right	383.69	91.82	467.25	84.62	10.933	**<0.0001**
		Left	357.13	97.64	419.35	92.24	3.672	**0.0079**
	Average power [W]	Right	72.13	17.76	85.55	16.63	9.695	**0.0000**
		Left	67.69	22.38	79.55	25.58	2.508	**0.0405**

Knee flexors								
Joint angle	Parameter	Leg	Before exercise		After exercise		Student's *t*-test	
			Mean	SD	Mean	SD	*t*	*P*

60°/s	Peak torque [N-m]	Right	34.80	7.80	40.36	6.74	3.341	**0.0124**
		Left	33.53	9.88	37.25	8.99	1.500	0.1774
	Peak torque/body weight [%]	Right	57.54	13.84	67.55	11.28	4.678	**0.0023**
		Left	58.09	18.19	63.63	15.39	1.344	0.2208
	Total work [J]	Right	188.25	48.42	237.53	41.56	4.137	**0.0044**
		Left	191.46	69.09	223.81	53.06	2.469	**0.0429**
	Average power [W]	Right	20.24	5.30	24.16	4.52	6.871	**0.0002**
		Left	19.79	7.78	22.73	6.33	1.846	0.1074

180°/s	Peak torque [N-m]	Right	26.20	4.91	31.38	7.36	2.815	**0.0260**
		Left	24.83	5.91	30.41	7.89	2.834	**0.0253**
	Peak torque/body weight [%]	Right	43.64	8.70	48.78	9.04	3.744	**0.0072**
		Left	41.18	12.17	45.95	12.49	4.779	**0.0020**
	Total work [J]	Right	235.31	54.22	280.59	67.94	5.006	**0.0016**
		Left	211.30	63.32	266.34	70.07	6.898	**0.0002**
	Average power [W]	Right	30.49	6.25	37.35	8.52	5.053	**0.0015**
		Left	27.96	8.48	35.29	8.86	8.909	**0.0000**

300°/s	Peak torque [N-m]	Right	25.61	3.93	26.48	4.42	1.842	0.1080
		Left	25.96	3.93	27.24	4.04	4.295	**0.0036**
	Peak torque/body weight [%]	Right	42.13	4.89	42.84	5.26	1.164	0.2826
		Left	42.11	6.80	43.88	6.50	4.760	**0.0021**
	Total work [J]	Right	154.54	48.26	175.24	56.40	2.604	**0.0352**
		Left	138.93	60.54	183.31	62.88	3.297	**0.0132**
	Average power [W]	Right	27.21	7.28	31.28	8.45	2.908	**0.0227**
		Left	23.84	10.01	31.99	9.53	4.289	**0.0036**

^*∗*^Significance of changes at level *P* < 0.05; *P* < 0.01; *P* < 0.001.

**Table 5:** Peak torque of flexor and extensor muscles of the knee joint at different angular velocities.

			Increase muscle strength						Friedman test	
Move	Parameter	Leg	60°/s		180°/s		300°/s			
			Mean	SD	Mean	SD	Mean	SD	*x* ^2^	*P* value
Extension	Peak torque [N-m]	Right	6.53	3.99	4.69	2.80	1.55	1.07	7.75	**0.021**
		Left	1.38	10.80	2.70	4.28	2.61	3.01	2.25	0.325
	Peak torque/body weight [%]	Right	18.31	11.57	9.10	3.69	1.30	1.18	10.75	**0.005**
		Left	10.84	22.75	4.68	6.66	1.08	7.21	9.00	**0.011**

Flexion	Peak torque [N-m]	Right	5.56	4.71	5.18	5.20	0.86	1.32	7.00	**0.030**
		Left	3.73	7.03	5.59	5.58	1.28	0.84	4.75	0.093
	Peak torque/body weight [%]	Right	10.01	6.05	5.14	3.88	0.71	1.73	10.75	**0.005**
		Left	5.54	11.65	4.78	2.83	1.76	1.05	3.25	0.197

**Table 6:** The coefficients of Spearman's-*ρ* rank correlation between Fullerton test results and peak torque value at angular velocities of 60°/s, 180°/s, and 300°/s in the group of studied women. Coefficients that were statistically significant at *P* < 0.05 were highlighted in bold.

Fullerton test	Peak torque PT [N-m]											
	Extensor	Extensor	Flexor	Flexor	Extensor	Extensor	Flexor	Flexor	Extensor	Extensor	Flexor	Flexor
	60 R	60 L	60 R	60 L	180 R	180 L	180 R	180 L	300 R	300 L	300 R	300 L
Eight foot up and go [s]	**−0.71**	**−0.66**	−0.64	−0.46	**−0.69**	**−0.66**	−0.38	−0.51	**−0.67**	**−0.65**	−0.35	−0.48
Chair stand [n]	**0.66**	**0.65**	0.35	0.42	**0.66**	**0.65**	0.26	0.35	**0.65**	**0.65**	0.24	0.31
Arm curl [n]	**0.65**	**0.66**	0.31	0.36	0.61	0.60	0.32	0.36	0.60	0.59	0.30	0.28
Chair seat and reach [cm]	0.17	0.31	0.26	0.31	0.13	0.03	0.16	0.14	0.10	0.05	0.20	0.16
Back scratch [cm]	0.15	0.11	0.22	0.14	0.06	0.09	0.12	0.21	0.05	0.03	0.1	0.03
6-minute walk test [m]	**0.71**	**0.65**	0.41	0.47	**0.69**	**0.65**	0.39	0.53	**0.67**	**0.65**	0.37	0.51

R—right, L—left.
